# Regulatory Mechanisms of Exogenous Gibberellin on Seed Germination and Transcriptomic Responses in *Lomatogonium rotatum*

**DOI:** 10.3390/genes16080878

**Published:** 2025-07-26

**Authors:** Kefan Cao, Yingtong Mu, Sihai Lu, Yanyan Zhao

**Affiliations:** 1 Key Laboratory of Grassland Resources of Ministry of Education, College of Grassland Science, Inner Mongolia Agricultural University, Hohhot 010018, China; caokefan1003@163.com (K.C.); myt100862@outlook.com (Y.M.); 2 National Engineering Research Center for Miniaturized Detection Systems, Northwest University, Xi’an 710069, China; 3 Institute of Basic Medical Sciences, Department of Basic Medical Sciences, Xi’an Medical University, Xi’an 710021, China

**Keywords:** *Lomatogonium rotatum*, Gibberellin (GA_3_), seed germination, transcriptome analysis, WGCNA

## Abstract

Gibberellins (GAs) are essential phytohormones that regulate seed dormancy release and germination. *Lomatogonium rotatum* (L.) Fries ex Nym is a traditional medicinal plant whose seed germination is often hindered by physiological dormancy. In this study, we systematically investigated the effects of exogenous GA_3_ on the seed germination of *L. rotatum* and elucidated the underlying molecular regulatory mechanisms via transcriptomic analysis. GA_3_ treatment (500 mg/L for 24 h) significantly improved the germination rate, vigor index, and other germination traits. RNA-seq analysis identified time-dependent transcriptional changes in GA_3_-treated seeds across three developmental stages (24 h, 72 h, and 96 h). KEGG enrichment and K-means clustering revealed dynamic actiSvation of hormonal signaling, secondary metabolism, and DNA replication pathways. WGCNA uncovered two hormone-responsive co-expression modules (Red and Lightcyan) corresponding to early and late stages of germination, respectively. Key genes related to ABA and GA biosynthesis and signal transduction showed phase-specific expression, highlighting the coordinated hormonal regulation during seed germination. Our findings provide new insights into the molecular basis of GA_3_-regulated seed germination and offer theoretical support for the cultivation and utilization of *L. rotatum*.

## 1. Introduction

*Lomatogonium rotatum* (L.) Fries ex Nym. is an annual herbaceous plant belonging to the genus *Lomatogonium* of the family Gentianaceae. This species is widely distributed across Asia, Europe, and North America, with significant populations in regions such as Inner Mongolia, Xinjiang, and Liaoning in China [1]. As an important component of traditional Mongolian medicine, *L. rotatum* exhibits notable therapeutic efficacy in the treatment of epidemics, typhoid fever, hepatic and biliary heat, and jaundice. The entire plant possesses medicinal value, particularly the roots, stems, leaves, and flowers, which contain swertiamarin, a compound shown to have significant hepatoprotective effects [2]. However, due to overharvesting and habitat destruction, wild populations of *L. rotatum* have sharply declined, highlighting the urgent need for artificial cultivation and ecological restoration. Although some studies on cultivation techniques and seedling propagation have emerged in recent years, systematic investigations into the seed morphology, dormancy mechanisms, and germination process of *L. rotatum* remain scarce, limiting the broader application and promotion of its artificial propagation [3].

Seed dormancy refers to the inability of viable seeds to germinate even under favorable environmental conditions, often caused by seed coat structure, incomplete embryo development, or the presence of endogenous inhibitory substances [4]. The release of seed dormancy is a critical step in plant growth and directly influences reproductive success and ecological adaptability [5]. Numerous studies have shown that exogenous gibberellins (GAs) can effectively break seed dormancy and promote germination [6,7]. For *L. rotatum*, seed germination rates are typically low, necessitating specific treatments such as GA soaking or cold stratification to improve germination performance [8].

Although studies specifically focused on *L*. *rotatum* remain limited, research on seed germination in other plant species provides important mechanistic insights. Gibberellins (GAs) have been widely demonstrated to promote seed germination by antagonizing abscisic acid (ABA) signaling and activating GA-responsive pathways. In crops such as rice, maize, and soybean, GA promotes radicle emergence by alleviating the inhibitory effects of DELLA proteins on downstream gene expression, thus initiating cell elongation and metabolic reactivation [9,10]. Furthermore, exogenous GA application has been shown to accelerate the transition from dormancy to germination in various species [11]. Given that germination involves a complex interplay of hormone biosynthesis, metabolism, and signaling, transcriptomic analysis of *L. rotatum* seeds under GA_3_ treatment is essential to uncover the molecular basis of its germination regulation.

This study aims to systematically investigate the morphological traits, germination characteristics, and molecular regulation of *L. rotatum* seeds, with a particular focus on the promotive effects of exogenous gibberellins. By utilizing transcriptomic techniques, we examined the gene expression changes in *L. rotatum* seeds treated with GA, with an emphasis on identifying differentially expressed genes associated with the ABA/GA signaling pathways. These findings are expected to provide a theoretical foundation for the ex situ cultivation under controlled greenhouse or field conditions, resource conservation, and medicinal development of *L. rotatum*. Additionally, the outcomes of this study may offer valuable references for understanding dormancy release and germination mechanisms in other plant species.

## 2. Materials and Methods

### 2.1. Plant Materials

The seeds of *L*. *rotatum* used in this study were collected in early October 2022 from a cultivated wild population located at the Xiumei Green Ecological Base (41.10° N, 110.12° E; elevation ~1350 m) in Wuchuan County, Inner Mongolia, China. The population was grown and maintained under semi-natural conditions to preserve genetic characteristics of native germplasm. Seed identification and authentication were carried out by the College of Grassland Science, Inner Mongolia Agricultural University. To ensure population-level representation and minimize maternal effect bias, seeds were harvested from at least 30 healthy, randomly selected individual plants. All seeds were pooled and thoroughly mixed to form a single experimental batch, which was used for all subsequent treatments to maintain experimental consistency.

### 2.2. Experimental Methods

#### 2.2.1. Determination of Seed Morphological Characteristics

The size and morphology of *L. rotatum* seeds were measured and observed using a Vernier caliper (Mitutoyo, Mitutoyo Corporation, Tokyo, Japan) and a stereomicroscope (Olympus, Olympus Corporation, Tokyo, Japan). A total of 100 seeds were randomly selected for measurement of their length (maximum longitudinal distance), width (maximum lateral distance), and thickness (maximum distance between dorsal and ventral surfaces). Seed coat color was recorded and categorized, and the proportion of seeds with each color was calculated.

#### 2.2.2. Seed Imbibition Rate

A total of 0.2 g of seeds were wrapped in filter paper and soaked in distilled water to assess imbibition rate. At 30 min intervals, seeds were removed, gently blotted to remove surface moisture, and weighed using an analytical balance with 0.1 mg precision. The seeds were then returned to distilled water for continued imbibition. This process was repeated until seed weight stabilized. The imbibition rate was calculated using the standard formula:

$$Imbibition\;Rate=\frac{Weight\;at\;time\;t-Initial\;weight}{Initial\;weight}\times 100$$

This measurement was repeated three times for each treatment group.

#### 2.2.3. Gibberellin Treatment

*L. rotatum* seeds were treated with gibberellic acid (GA_3_) to assess its effects on germination. Two independent experiments were conducted. In the first experiment, seeds were soaked for 24 h in GA_3_ solutions of varying concentrations (100, 300, 400, 450, 500, 550, and 700 mg/L). In the second experiment, the optimal concentration identified from the first experiment (500 mg/L) was used to investigate the effect of different soaking durations (12, 15, 18, 21, 24, and 48 h). Each treatment consisted of three biological replicates with 100 seeds per replicate. Following GA_3_ treatment, seeds were incubated in a climate-controlled growth chamber under alternating temperatures of 20 °C (night, 20:00–08:00) and 25 °C (day, 08:00–20:00), with a 12 h light/12 h dark photoperiod. The light intensity during the photoperiod was maintained at approximately 120 μmol·m^−2^·s^−1^ using white LED lights. These environmental conditions were consistently applied across all treatments to ensure experimental reproducibility.

#### 2.2.4. Transcriptome Sequencing and Analysis

To investigate the molecular regulatory mechanisms underlying GA_3_-mediated germination in *L. rotatum* seeds, transcriptome sequencing was performed at three time points: 24 h (T1), 72 h (T2), and 96 h (T3) after treatment. Prior to transcriptome sampling, seeds were pre-treated by soaking in 500 mg/L GA_3_ for 24 h and then transferred to a climate-controlled chamber (25 °C day/20 °C night, 12 h photoperiod, 120 μmol·m^−2^·s^−1^ light intensity). Control groups were treated with distilled water under the same conditions. These three time points were selected to capture early signaling events (T1), peak transcriptional activity and radicle emergence (T2), and sustained physiological activity in ungerminated seeds (T3).

Importantly, for each time point, only non-germinated seeds—i.e., seeds that had not undergone visible radicle protrusion—were sampled. This was done to focus the analysis on internal transcriptional regulation rather than post-germination processes. To ensure the biological activity and viability of these non-germinated seeds at 96 h (T3), embryo developmental integrity was assessed using paraffin sectioning and light microscopy (see Section 2.2.5), confirming the presence of morphologically complete embryos in most seeds (>80%) with evidence of continued growth and differentiation. Moreover, TTC staining confirmed that the majority of ungerminated seeds at 96 h were still viable.

For each treatment and time point, 0.2 g of seeds (approximately 200–250 seeds) were collected and pooled from three biological replicates. All samples were immediately frozen in liquid nitrogen and stored at −80 °C until RNA extraction.

Total RNA was extracted using TRIzol reagent (Invitrogen, Waltham, MA, USA), and RNA quality was verified using an Agilent 2100 Bioanalyzer (Agilent Technologies, Santa Clara, CA, USA) with RIN ≥ 7.0. Library construction was performed using a NEBNext^®^ Ultra™ RNA Library Prep Kit (New England Biolabs, Ipswich, MA, USA), and sequencing was conducted on a Illumina NovaSeq 6000 platform (paired-end, 150 bp reads) by Biomarker Technologies (Beijing, China).

Raw reads were quality-filtered using fastp v0.23.2, and clean reads were aligned to the de novo transcriptome assembled by Trinity v2.13.2. Transcript quantification was performed using Salmon v1.10.1, and differential expression analysis was carried out using DESeq2 v1.36.0. Genes with FDR < 0.05 and |log_2_FC| ≥ 1 were considered significantly differentially expressed. Functional enrichment analysis (GO, KEGG) was performed using clusterProfiler v4.4.4, and gene co-expression networks were constructed using WGCNA v1.71.

#### 2.2.5. Microscopic Observation of Embryo Development

To observe the progression of embryo development, seeds of *L. rotatum* were subjected to 500 mg/L GA_3_ treatment for 24 h, then incubated at 20/25 °C (12 h light/12 h dark, 120 μmol·m^−2^·s^−1^) for up to 72 h. Samples were collected at 0 h (untreated), 36 h, 48 h, and 72 h post-incubation. At each time point, 30 seeds were randomly selected and fixed in FAA solution (formalin/acetic acid/ethanol = 5:5:90, *v*/*v*/*v*) for 24 h. After fixation, seeds were dehydrated through a graded ethanol series, cleared in xylene, embedded in paraffin, and sectioned at 10 μm thickness. Sections were stained with safranin and fast green, and then observed and photographed using a light microscope (Olympus BX53, Olympus Corporation, Tokyo, Japan). Embryo formation rate and developmental stage (globular, heart-shaped, torpedo) were recorded.

#### 2.2.6. Calculation of Germination Parameters

Four germination parameters were evaluated to assess seed germination performance: germination percentage, germination potential, germination index, and vigor index.

$$\mathrm{Germination}\;\mathrm{percentage}\;(\%):\;Germination\;percentage=\left(\frac{Number\;of\;germinated\;seeds}{Total\;number\;of\;seeds}\right)\times 100$$

Germination potential (%): The percentage of seeds that germinated within the first 5 days of incubation relative to the total number of seeds.

$$\mathrm{Germination}\;\mathrm{index}\;(\mathrm{GI}):\;GI=\sum \left(\frac{Gt}{Dt}\right)$$

Vigor index (VI): VI=Germination percentage×Mean seedling length (cm)

Mean seedling length was determined by measuring the average total length (root + shoot) of ten randomly selected seedlings from each treatment on day 10.

All measurements were performed in three biological replicates (n = 100 seeds per replicate), and standard deviations were calculated.

#### 2.2.7. qRT-PCR Validation

To validate the reliability of the RNA-seq results, 15 differentially expressed genes (DEGs) involved in GA and ABA metabolic and signaling pathways were randomly selected for quantitative real-time PCR (qRT-PCR) analysis. Gene-specific primers were designed using Primer Premier 5.0 based on transcript sequences obtained from the de novo assembly results of *L. rotatum* generated in this study.

The reference gene *Actin* was selected as the internal control to normalize gene expression levels, based on its stable expression across all transcriptome samples [12]. Primer sequences for target and reference genes are listed in Supplementary Table S1.

Total RNA used for qRT-PCR was the same as that used for transcriptome sequencing. First-strand cDNA was synthesized using the PrimeScript RT reagent Kit with gDNA Eraser (Takara, Kusatsu, Japan) according to the manufacturer’s instructions.

qRT-PCR was performed using TB Green^®^ Premix Ex Taq™ II (Takara, Kusatsu, Japan) on a Bio-Rad CFX96 Real-Time PCR System. The 20 μL reaction mixture contained:

- 10 μL TB Green Premix.
- 1 μL forward primer (10 μM).
- 1 μL reverse primer (10 μM).
- 1 μL cDNA template.
- 7 μL RNase-free H_2_O.

Thermal cycling conditions were as follows: 95 °C for 30 s, followed by 40 cycles of 95 °C for 5 s and 60 °C for 30 s. A melt curve analysis was performed at the end of the amplification to confirm the specificity of the PCR products.

Relative expression levels were calculated using the 2^−ΔΔCt^ method. All reactions were performed in three biological replicates, and the results were expressed as mean ± standard deviation (SD).

#### 2.2.8. Statistical Analysis

All phenotypic data (e.g., germination percentage, germination index, vigor index) were analyzed using one-way analysis of variance (ANOVA), followed by Duncan’s multiple range test to determine significant differences among treatment groups. Statistical significance was assessed at *p* < 0.05. All analyses were performed using SPSS Statistics software (version 26.0; IBM Corp., Armonk, NY, USA), and results are presented as mean ± standard deviation (SD) based on three biological replicates (n = 100 seeds per replicate).

## 3. Results

### 3.1. Seed Size, Morphology, and Structure of L. rotatum

#### 3.1.1. Seed Morphological Characteristics of *L. rotatum*

**Figure 1:**
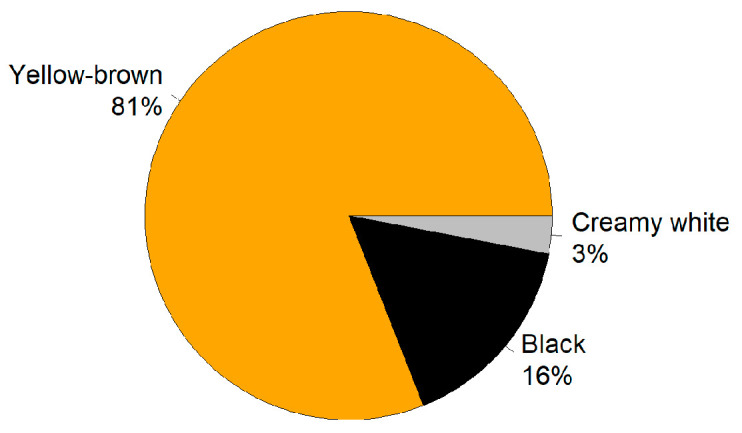
Color distribution of *L. rotatum* seeds. Yellow-brown seeds accounted for 81%, followed by black seeds (16%) and creamy white seeds (3%).

The morphological analysis of *L. rotatum* seeds revealed that the average seed length was 0.450 ± 0.065 mm, width was 0.330 ± 0.068 mm, and thickness was 0.280 ± 0.074 mm. The thousand-seed weight was 0.063 ± 0.006 g. In terms of color distribution, the majority of seeds were yellowish-brown (81%), followed by black (16%) and creamy white (3%) (Figure 1).

#### 3.1.2. Embryo Morphogenesis Under Gibberellin Treatment

The seeds of *L. rotatum* are spherical with an uneven surface, consisting of a seed coat, embryo, and endosperm. The embryo is located in the central-lower region of the seed and appears colorless and transparent. Due to the biological characteristics of wild germplasm, the seeds of *L. rotatum* often exhibit incomplete embryonic development upon detachment from the mother plant. In untreated seeds, the embryo formation rate is approximately 20%, and the embryos are predominantly globular in shape.

**Figure 2:**
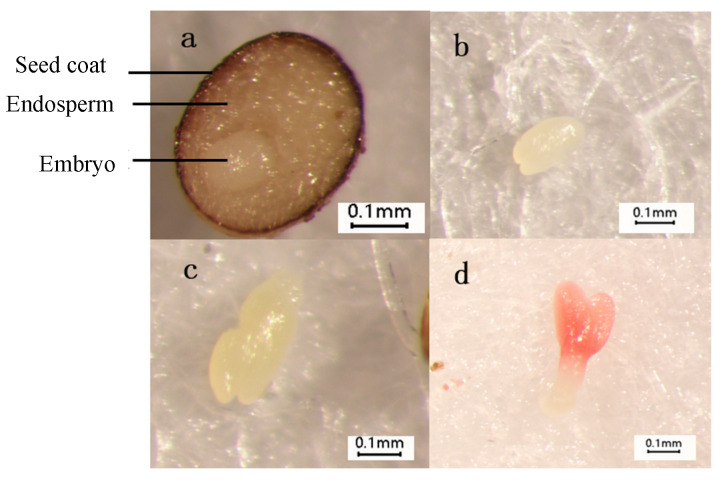
Morphological changes of *L. rotatum* embryo: (**a**) Spherical embryo; (**b**) Early heart-shaped embryo; (**c**) Heart-shaped embryo; (**d**) Torpedo-shaped embryo.

After soaking in 500 mg/L gibberellic acid (GA_3_) for 24 h and incubating at 20/25 °C for 36 h, the embryo formation rate increased to about 40%, with the embryos gradually developing into the early heart-shaped stage. Following 48 h of incubation, the rate reached 55%, and the two cotyledon primordia became more distinct, entering the heart-shaped embryo stage. At 72 h, the embryo formation rate further increased to 75%, during which the apical cotyledon primordia continued to differentiate and progressed toward the torpedo-shaped stage (Figure 2).

#### 3.1.3. Seed Water Uptake Capacity

**Figure 3:**
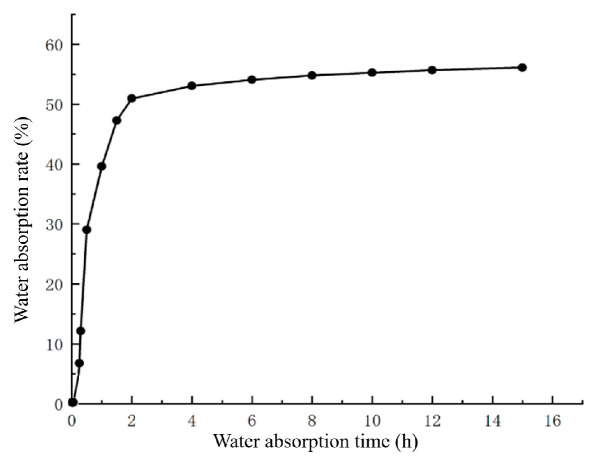
Water absorption curve of *L. rotatum* seeds.

To investigate the water absorption characteristics of *L*. *rotatum* seeds, the changes in seed water content over time were measured, and a water uptake curve was plotted (Figure 3). The results revealed that the water absorption process of *L. rotatum* seeds could be divided into three distinct phases. The first phase was a rapid water uptake stage (first 4 h), during which the seeds absorbed water rapidly, reaching a water absorption rate of 53% at 4 h. During this period, imbibition was mainly driven by the seed’s imbibitional force. The second phase was a slow water uptake stage (4 to 12 h), where the rate of water absorption gradually decreased and approached a plateau, nearing saturation. The third phase was the saturation stage, in which the seeds reached their maximum water absorption and entered a plateau phase with no further increase (Figure 3).

Overall, no physical barriers to water uptake were observed in *L. rotatum* seeds throughout the process, indicating that the seed coat exhibits good permeability and is not a limiting factor for germination.

### 3.2. Effects of Gibberellic Acid Treatment on Seed Germination of L. rotatum

To investigate the influence of GA_3_ on seed germination, we performed two independent experiments: (1) a concentration screening experiment with fixed soaking duration (24 h), and (2) a soaking time optimization experiment using the most effective concentration (500 mg/L) determined from the first test.

#### 3.2.1. Effects of Different GA_3_ Concentrations on Seed Germination

**Figure 4:**
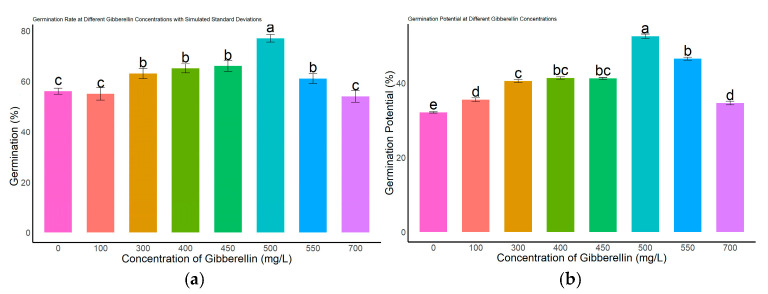

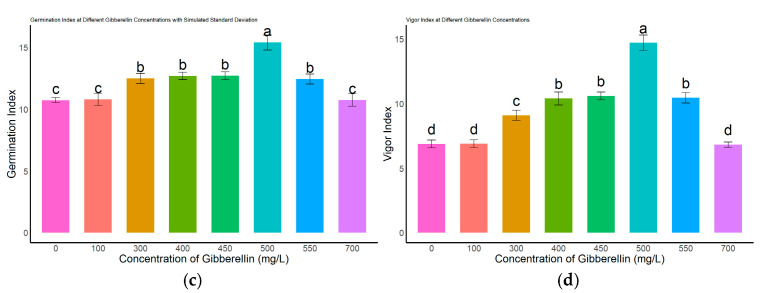
Effects of different GA_3_ concentrations on seed germination of *L. rotatum*: (**a**) Germination rate under different GA_3_ concentrations; (**b**) Germination potential under different GA_3_ concentrations; (**c**) Germination index under different GA_3_ concentrations; (**d**) Vigor index under different GA_3_ concentrations. Bars represent the mean ± standard deviation (SD). Lowercase letters above the bars indicate statistical significance determined by one-way ANOVA followed by post-hoc Tukey’s test (*p* < 0.05). Treatments with the same letter are not significantly different, and treatments with different letters are significantly different.

Different concentrations of gibberellic acid (GA_3_) had significant effects on the germination performance of *L*. *rotatum* seeds (Figure 4). Regarding the germination rate (Figure 4a), no significant differences were observed among the 300, 400, 450, and 550 mg/L treatments (*p* > 0.05), with germination rates ranging from 63.2% to 68.4%. However, these were all significantly higher than those of the control (0 mg/L), as well as the 100 mg/L and 700 mg/L treatment groups (*p* < 0.05). Regarding germination potential (Figure 4b), the 500 mg/L GA_3_ treatment group exhibited the highest value, reaching 47.3%, which was significantly greater than that of all other treatment groups and the control (*p* < 0.05). Germination index analysis (Figure 4c) showed that the 500 mg/L treatment group had the highest index of 15.55, significantly exceeding other treatments and clearly higher than the control value of 10.4. In terms of vigor index (Figure 4d), the 500 mg/L group also achieved the highest value of 13.86, which was significantly higher than all other concentrations and the control (*p* < 0.05).

Taken together, these results indicate that 500 mg/L GA_3_ is the optimal concentration for promoting seed germination in *L. rotatum*, showing the best performance across all germination parameters.

#### 3.2.2. Effects of Different Soaking Durations on Seed Germination of *L. rotatum*

**Figure 5:**
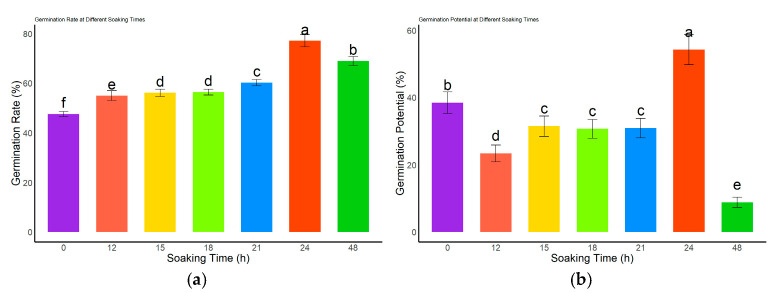

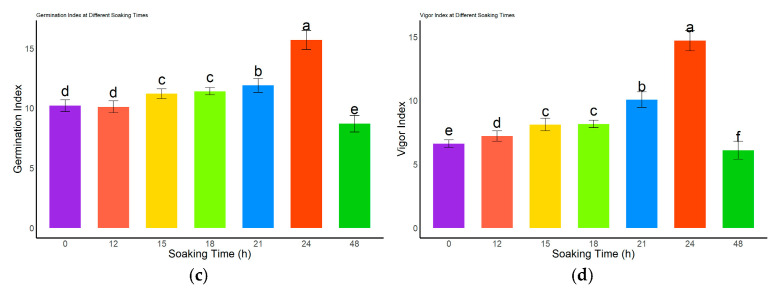
Effects of different GA_3_ soaking durations on seed germination of *L. rotatum*: (**a**) Germination rate under different soaking times; (**b**) Germination potential under different soaking times; (**c**) Germination index under different soaking times; (**d**) Vigor index under different soaking times. Bars represent the mean ± standard deviation (SD). Lowercase letters above the bars indicate statistical significance determined by one-way ANOVA followed by post-hoc Tukey’s test (*p* < 0.05). Treatments with the same letter are not significantly different, and treatments with different letters are significantly different.

The germination performance of *L*. *rotatum* seeds treated with 500 mg/L GA_3_ was significantly affected by soaking duration (Figure 5). The germination rate gradually increased with extended soaking time and peaked at 77.25% after 24 h, which was significantly higher than all other treatments and the control (*p* < 0.05). However, the germination rate declined following 48 h of soaking (Figure 5a). In terms of germination potential (Figure 5b), no significant differences were observed among the 15, 18, and 21 h treatments (*p* > 0.05), whereas the lowest germination potential (3.5%) was recorded at 48 h. As shown in Figure 5c, the germination index of the 12 h soaking treatment did not significantly differ from that of the control (*p* > 0.05), but was significantly lower than those of all other treatment durations (*p* < 0.05). The vigor index analysis (Figure 5d) revealed that the 24 h treatment resulted in the highest vigor index, significantly higher than all other treatments and the control (*p* < 0.05), while the 48 h treatment yielded the lowest vigor index. Collectively, these results indicate that 24 h is the optimal soaking duration for GA_3_ treatment of *L. rotatum* seeds.

### 3.3. RNA-Seq Analysis of Gibberellin-Induced Seed Germination

#### 3.3.1. Quality Assessment of Sequencing Data

To comprehensively analyze transcriptomic changes regulated by gibberellic acid (GA_3_) during the seed germination of *L*. *rotatum*, a total of 18 samples were subjected to high-throughput RNA sequencing. These included biological replicates from both GA_3_-treated groups (24 h, 72 h, and 96 h) and H_2_O-treated controls at the corresponding time points. The number of clean reads per sample ranged from 19,221,648 to 22,168,127, with corresponding base counts between 5.75 and 6.63 Gb, meeting the requirements for downstream analyses (Table 1).

In terms of sequencing quality, all samples achieved a Q30 score above 93.9%, with the highest reaching 94.89%, indicating high sequencing accuracy and reliability. GC content ranged from 42.55% to 43.90%, which falls within the expected range for plant transcriptomes, suggesting that the samples were free from significant contamination and the library construction quality was satisfactory.

#### 3.3.2. Sample Correlation and Principal Component Analysis (PCA)

**Figure 6:**
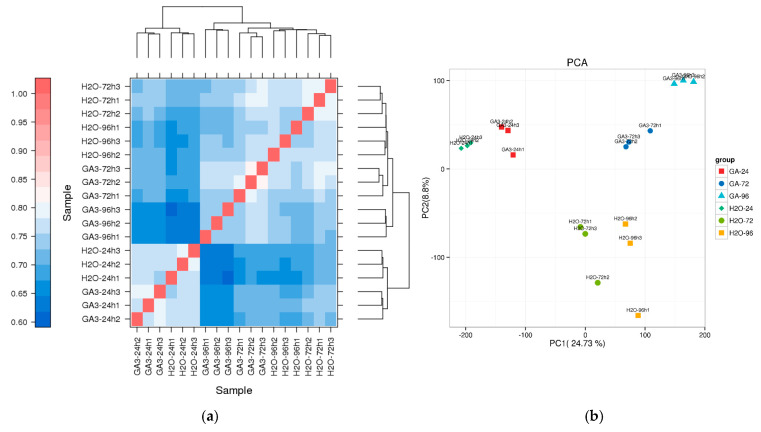
Sample correlation and PCA analysis of transcriptome data in *L. rotatum* under different GA_3_ treatments: (**a**) Hierarchical clustering heatmap based on Pearson correlation coefficients among 18 samples, including three time points (24 h, 72 h, 96 h) for both GA_3_-treated and H_2_O control groups; (**b**) Principal component analysis (PCA) showing sample distribution in two-dimensional space, where PC1 and PC2 account for 24.73% and 8.8% of the variation, respectively, indicating distinct gene expression profiles between treatments and time points.

To evaluate the overall consistency of gene expression among samples, Pearson correlation coefficients were used to generate a hierarchical clustering heatmap. The results showed high correlation among biological replicates within each treatment group, with correlation coefficients generally exceeding 0.9, indicating good reproducibility and reliable data quality. Notably, the GA_3_-96 h group exhibited the tightest clustering and the highest internal consistency. The GA_3_-24 h and H_2_O-24 h groups formed separate branches, suggesting that GA_3_ treatment had a distinct impact on transcriptional expression at an early stage (Figure 6a).

Additionally, PCA revealed that the first principal component (PC1) and the second principal component (PC2) explained 24.73% and 8.8% of the total expression variance, respectively. Samples from different treatment groups were clearly separated in the two-dimensional PCA space, with *GA_3_*-96 h, *GA_3_*-72 h, and *GA_3_*-24 h samples distinctly separated from their corresponding H_2_O controls. This indicates that transcriptional expression patterns varied significantly under different treatment conditions, and that *GA_3_* regulation of seed germination in *L. rotatum* exhibits a time-dependent pattern (Figure 6b).

#### 3.3.3. Differentially Expressed Gene (DEG) Statistics

**Figure 7:**
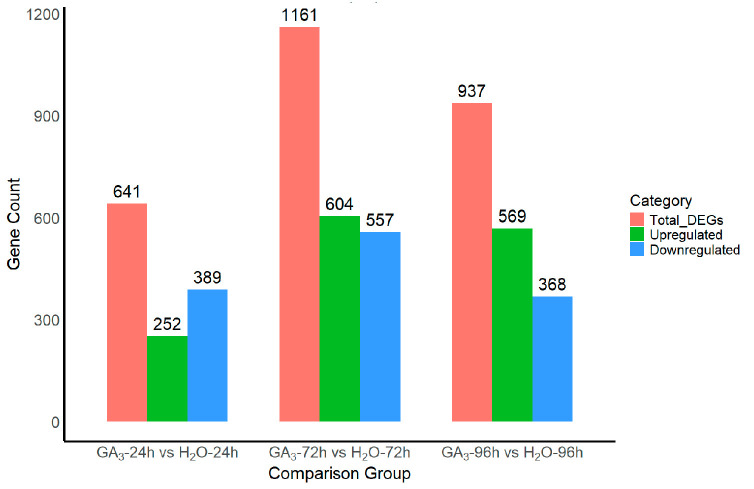
Differentially expressed gene (DEG) statistics between GA3-treated and H2O control samples at different time points.

To investigate the transcriptional regulatory effects of GA_3_ on *L*. *rotatum* seeds, differential expression analysis was conducted between GA_3_-treated and H_2_O control groups at three time points (24 h, 72 h, and 96 h). A total of 641 differentially expressed genes (DEGs) were identified between the GA_3_-24 h and H_2_O-24 h samples, including 252 upregulated and 389 downregulated genes. In the GA_3_-72 h vs. H_2_O-72 h comparison, 1161 DEGs were detected, with 604 upregulated and 557 downregulated genes, representing the largest number of DEGs among the three time points. For the GA_3_-96 h vs. the H_2_O-96 h group, 937 DEGs were identified, comprising 569 upregulated and 368 downregulated genes. The overall trend indicates that GA_3_ treatment induced pronounced transcriptional changes in seeds, with the number of DEGs increasing over time and peaking at 72 h, followed by a slight decrease at 96 h. This suggests that the transcriptional regulation exerted by GA_3_ during seed germination is most significant at the 72 h stage (Figure 7).

Figure 7**.** Differentially expressed gene (DEG) statistics between GA_3_-treated and H_2_O control samples at different time points.

#### 3.3.4. KEGG Pathway Enrichment Analysis of Differentially Expressed Genes

**Figure 8:**
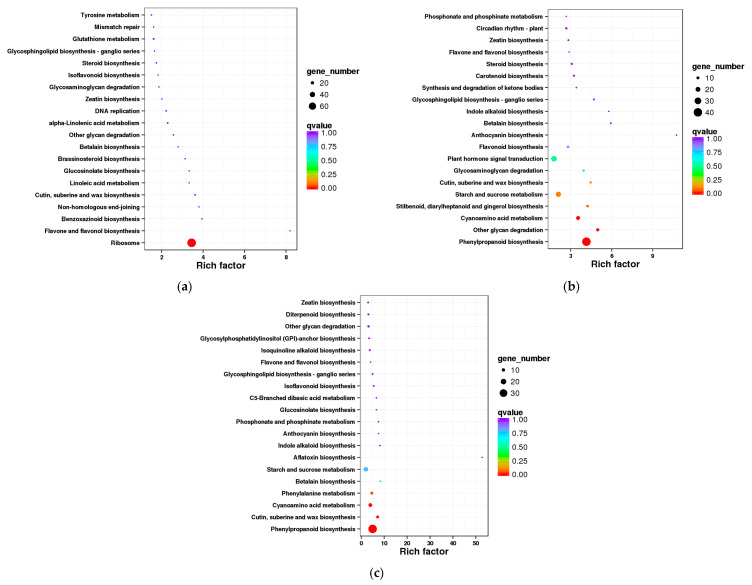
KEGG pathway enrichment analysis of differentially expressed genes in *L. rotatum* seeds under GA_3_ treatment at different time points: (**a**) Enrichment in the GA_3_-24 h vs. H_2_O-24 h comparison; (**b**) Enrichment in the GA_3_-72 h vs. H_2_O-72 h comparison; (**c**) Enrichment in the GA_3_-96 h vs. H_2_O-96 h comparison. Bubble size indicates the number of genes in each pathway, and color gradient reflects statistical significance (−log_10_(*P*)), with darker colors representing higher significance.

To further explore the metabolic pathway changes in *L*. *rotatum* seeds in response to gibberellin (GA_3_) treatment during germination, KEGG pathway enrichment analysis was conducted on differentially expressed genes (DEGs) from each treatment group. In the GA_3_-24 h vs. H_2_O-24 h comparison group, significantly enriched pathways included ribosome, flavonoid, and flavonol biosynthesis, benzoxazinoid biosynthesis, and phenylpropanoid biosynthesis. Among them, the “ribosome” pathway showed the highest rich factor and the lowest *q*-value, indicating that GA_3_ significantly regulates the expression of protein translation-related genes during early germination, potentially accelerating embryonic cell growth (Figure 8a).

In the *GA_3_-72 h* vs. *H_2_O-72 h* comparison, DEGs were primarily enriched in phenylpropanoid biosynthesis, secondary metabolite biosynthesis (such as plant hormone signal transduction, flavonoid, and lignin biosynthesis), and carbohydrate metabolism (including starch and sucrose metabolism). Notably, the “phenylpropanoid biosynthesis” and “plant hormone signal transduction” pathways were significantly enriched, suggesting that GA_3_ treatment at this stage activates transcriptional networks related to cell wall modification, signal transduction, and secondary metabolism, thereby promoting hypocotyl elongation and tissue differentiation (Figure 8b).

In the *GA_3_-96 h* vs. *H_2_O-96 h* group, DEGs were significantly enriched in phenylpropanoid biosynthesis, linoleic acid metabolism, phenylalanine metabolism, flavonoid biosynthesis, and cell wall-related pathways such as cutin and wax biosynthesis. Particularly, the “phenylpropanoid biosynthesis” pathway was the most significantly enriched, indicating that in the later stage of germination, GA_3_ continues to regulate cell wall-related pathways involved in root embryo development and seed coat penetration (Figure 8c).

#### 3.3.5. Trend Analysis of Differentially Expressed Genes

**Figure 9:**
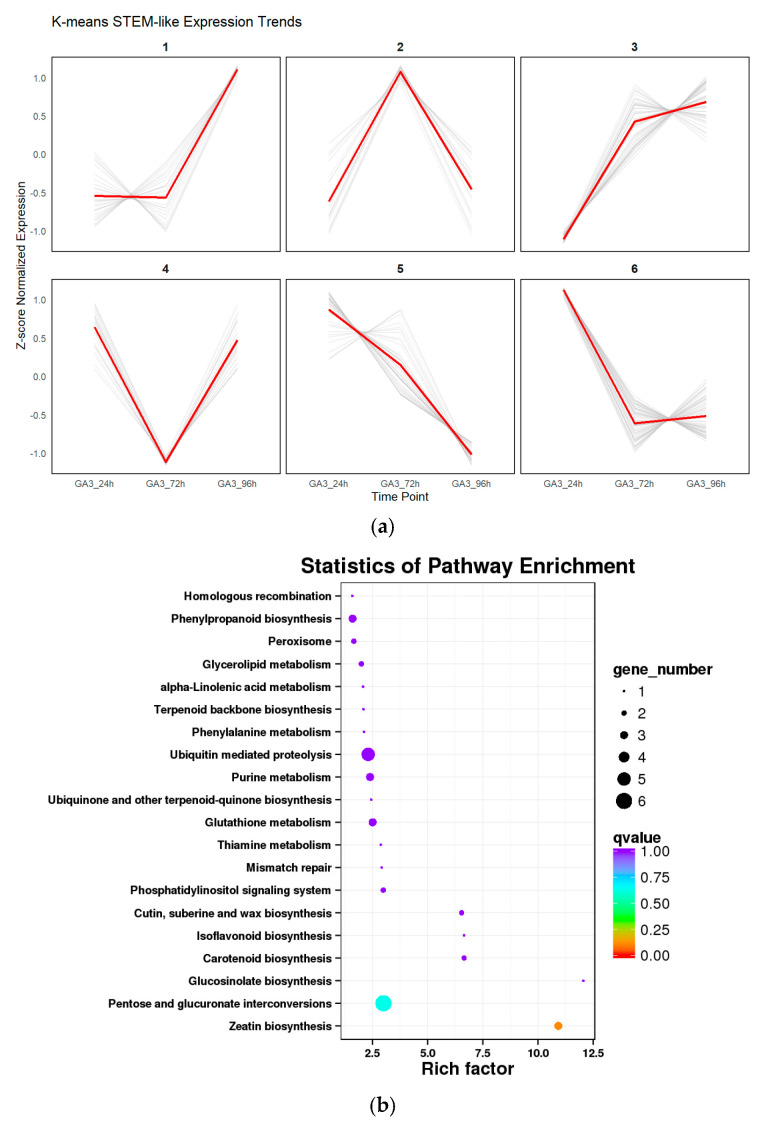
Trend clustering and KEGG enrichment analysis of differentially expressed genes: (**a**) K-means clustering divided DEGs in *L. rotatum* seeds under different *GA_3_* treatment durations into six expression patterns (Cluster 1–6), showing dynamic transcriptional responses over time; (**b**) KEGG enrichment bubble plot for each expression cluster, illustrating the significantly enriched metabolic and signaling pathways. Rich factor indicates enrichment strength, bubble size represents gene count, and color denotes –log_10_(*q*-value).

To explore the dynamic expression patterns of differentially expressed genes (DEGs) under *GA_3_* treatment, all DEGs were standardized and subjected to K-means clustering analysis. Six expression clusters (Cluster 1–6) were ultimately identified, each exhibiting distinct temporal expression trends (Figure 9a). Genes in Cluster 1 and Cluster 3 showed a continuously increasing trend, suggesting their potential involvement in *GA_3_*-induced positive regulatory pathways. In contrast, genes in Cluster 5 showed a persistent downward trend, indicating possible roles in inhibitory responses or negative feedback regulation. Cluster 2 and Cluster 4 displayed “up–down” and “down–up” expression patterns, respectively, implying that they may be critical during the early or late stages of treatment. Cluster 6 showed a typical V-shaped pattern, possibly reflecting rapid early responses followed by recovery mechanisms.

Further KEGG pathway enrichment analysis of genes within each cluster revealed significant enrichment in various metabolic and signaling pathways (Figure 9b), including zeatin biosynthesis, isoflavonoid and carotenoid metabolism, glutathione metabolism, and ubiquinone and terpenoid-quinone biosynthesis. These pathways are closely related to hormone regulation, antioxidant activity, and secondary metabolism. Notably, the enrichment of hormone-related pathways in Cluster 2 and Cluster 3 highlights the extensive remodeling of endogenous hormone regulatory networks under GA_3_ treatment. Meanwhile, the enrichment of primary metabolic pathways in Cluster 5 indicates that certain basic physiological processes may be suppressed during treatment. Overall, the combination of trend clustering and pathway enrichment analysis revealed not only time-dependent transcriptional responses induced by *GA_3_*, but also the complex regulatory mechanisms through which *GA_3_* modulates hormone biosynthesis, signal transduction, and metabolic networks.

### 3.4. WGCNA Analysis

#### 3.4.1. Construction of Weighted Gene Co-Expression Network and Identification of Key Modules

To further identify functional modules closely related to *GA_3_*-induced seed germination in *L*. *rotatum*, a weighted gene co-expression network analysis (WGCNA) was conducted using the gene expression data from all samples. After filtering and preprocessing the expression matrix, hierarchical clustering was performed using the dynamic tree cut method, and a total of 30 representative co-expression modules were identified. Each module eigengene was then correlated with six different treatment conditions (*GA_3_*-24 h, *GA_3_*-72 h, *GA_3_*-96 h, and their corresponding controls) via Pearson correlation analysis to uncover modules significantly associated with treatment time and hormone induction.

**Figure 10:**
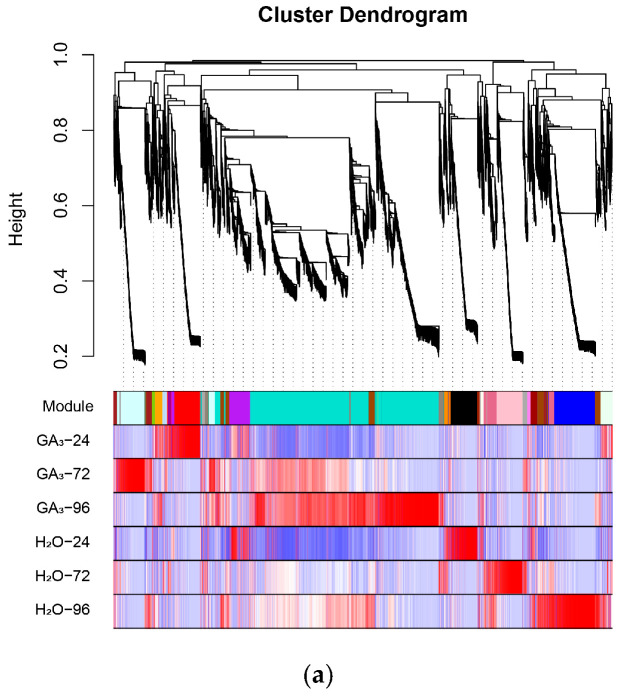

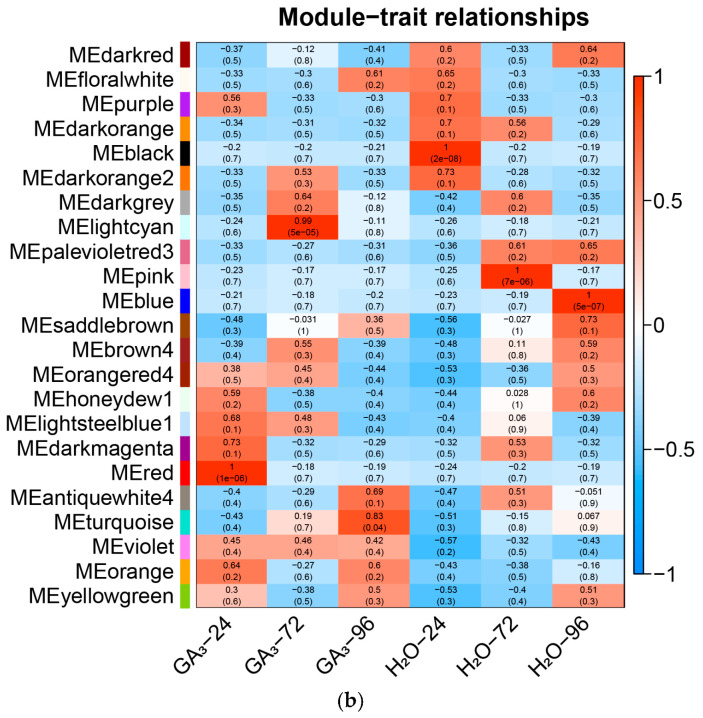
Construction of co-expression network and identification of key modules using WGCNA: (**a**) Dendrogram of gene clustering and module assignment across different treatment conditions in *L. rotatum*. Each branch represents a gene, and each color represents a distinct co-expression module; (**b**) Module–trait relationships showing Pearson correlation coefficients between module eigengenes and sample traits (treatment × time).

The results revealed that multiple modules were significantly correlated with *GA_3_* treatment. In particular, the Red module showed a highly significant positive correlation with the *GA_3_*-24 h group (r = 1, *p* = 1 × 10^−6^), indicating that genes in this module may play vital roles during the early phase of *GA_3_* response, including hormone signal perception, initial transcriptional activation, and the initiation of cellular activity. The Lightcyan module exhibited the strongest positive correlation with the *GA_3_*-72 h group (r = 0.99, *p* = 5 × 10^−5^), suggesting its involvement in physiological processes occurring during the middle-to-late stages of germination, such as energy metabolism, endosperm mobilization, and cell division (Figure 10a).

Furthermore, the module–trait correlation heatmap demonstrated clear differences in expression patterns across treatment samples, and genes within each module exhibited highly consistent expression trends, indicating stable and coherent regulatory mechanisms (Figure 10b). Based on the high correlation and statistical significance, the Red and Lightcyan modules were ultimately identified as the key co-expression modules in this study, representing the core transcriptional responses during early and middle-to-late stages of *GA_3_*-induced seed germination, respectively. These results lay a solid foundation for subsequent functional annotation, enrichment analysis, and identification of key regulatory genes.

#### 3.4.2. Identification and Functional Enrichment Analysis of Key Modules

To identify core functional modules closely associated with *GA_3_*-induced seed germination, a weighted gene co-expression network was constructed using WGCNA. Among the modules identified, the Red and Lightcyan modules exhibited the most significant correlations with *GA_3_* treatment.

**Figure 11:**
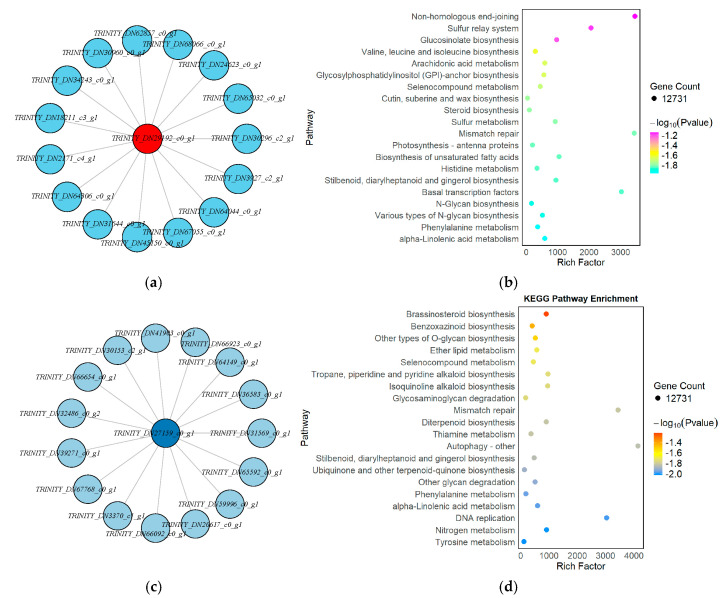
KEGG enrichment and hub gene network analysis of key WGCNA modules associated with GA_3_-induced seed germination in *L. rotatum*: (**a**) Hub gene network of the Red module; (**b**) KEGG pathway enrichment of the Red module; (**c**) Hub gene network of the Lightcyan module; (**d**) KEGG pathway enrichment of the Lightcyan module.

The Red module was strongly positively correlated with the *GA_3_*-24 h samples (correlation coefficient = 1, *p* = 1 × 10^−6^), suggesting that its constituent genes may be involved in early transcriptional responses to gibberellin signaling. Network visualization further revealed that *TRINITY_DN29192_c0_g1* served as the central hub gene in this module, with the highest degree of connectivity, indicating its potential regulatory role during the initial stage of *GA_3_* perception and signaling (Figure 11a). KEGG enrichment analysis indicated that genes in the Red module were significantly enriched in pathways such as sulfur metabolism, steroid biosynthesis, photosynthesis-antenna proteins, and non-homologous end joining. These pathways are likely to play essential roles in *GA_3_*-mediated signal transduction and cellular reactivation during the early transition from dormancy to germination (Figure 11b).

In contrast, the Lightcyan module was highly positively correlated with the *GA_3_*-72 h samples (correlation coefficient = 0.99, *p* = 5 × 10^−5^), indicating that the genes in this module may be functionally relevant to mid-to-late-stage physiological processes such as energy metabolism, cell expansion, and endosperm mobilization. The hub gene *TRINITY_DN27159_c0_g1* formed a radial interaction network with numerous key genes, underscoring its central regulatory role in this module (Figure 11c). KEGG enrichment analysis revealed that genes in the Lightcyan module were involved in pathways such as brassinosteroid biosynthesis, benzoxazinoid biosynthesis, O-glycoside biosynthesis, ether lipid metabolism, and autophagy. Additionally, the module was enriched in cell cycle–related pathways such as mismatch repair and DNA replication, suggesting heightened metabolic activity and entry into mitotic division at 72 h post-treatment (Figure 11d).

In summary, the Red and Lightcyan modules correspond to the early and mid-to-late transcriptional regulatory phases of *GA_3_*-induced seed germination, respectively. The identification of hub genes and significantly enriched pathways within these modules provides key molecular insights into how *GA_3_* modulates dormancy release and the initiation of germination in *L*. *rotatum*.

### 3.5. Expression Patterns of Hormone Biosynthesis and Signal Transduction Genes

#### 3.5.1. Expression of ABA-Related Biosynthesis and Signal Transduction Genes

Abscisic acid (ABA) plays a central role in regulating seed dormancy and germination. In this study, transcriptome data were used to analyze the expression patterns of ABA biosynthesis and signaling-related genes in *L*. *rotatum* seeds at different germination stages (T1, T2, and T3). A total of 23 differentially expressed genes (DEGs) associated with ABA were identified, and their expression dynamics are shown in the corresponding figures.

In the ABA biosynthesis pathway, *L. rotatum* synthesizes ABA primarily through the indirect C40 carotenoid pathway. Among the four DEGs encoding zeaxanthin epoxidase (*ZEP*), a key enzyme in this pathway, two were downregulated at the T2 stage and the other two were significantly upregulated at T3, suggesting a time-dependent regulatory role of *ZEP*. For 9-cis-epoxycarotenoid dioxygenase (*NCED*), a rate-limiting enzyme, three out of four DEGs were downregulated at T1 and T2, while only one was upregulated at T2, indicating strong suppression of ABA synthesis during early germination. Downstream metabolic enzymes such as *ABA2* and *AOG* were also downregulated at T2, further confirming reduced ABA biosynthesis during mid-germination.

**Figure 12:**
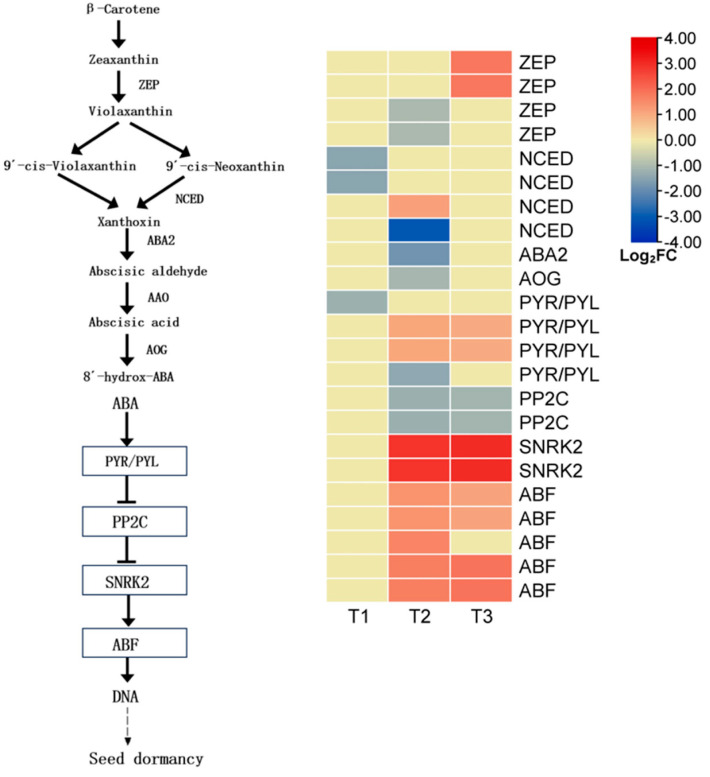
Expression profiles of key genes involved in abscisic acid (ABA) biosynthesis and signal transduction during *L. rotatum* seed germination.

In the ABA signaling pathway, ABA binds to its receptor *PYR/PYL*, which inhibits the negative regulator *PP2C*, thereby activating downstream protein kinases *SnRK2*, and ultimately regulating the expression of germination-responsive genes via the transcription factor *ABF*. In this study, four DEGs related to *PYR/PYL* were identified: one was downregulated at T1, another at T2, and the remaining two were upregulated at T3, suggesting that ABA signal perception was suppressed during early-to-mid-germination but restored at the late stage. Two *PP2C* DEGs showed stable expression at T3, likely due to upstream *PYR/PYL*-mediated inhibition. Two *SnRK2* and five *ABF*-related DEGs were significantly upregulated at T3, indicating enhanced activation of ABA-responsive transcription factors and promoting the expression of downstream genes involved in germination (Figure 12).

Overall, *L. rotatum* seeds exhibited a general downregulation in ABA biosynthesis during the germination process, while downstream kinases and response elements in the signaling pathway were activated in the later stages. This suggests a finely tuned ABA regulatory network: ABA content is reduced in the early phase to release dormancy, and its signaling components are activated in the middle-to-late phases to coordinate embryonic growth and germination.

#### 3.5.2. Expression of Genes Related to GA Metabolism and Signal Transduction

**Figure 13:**
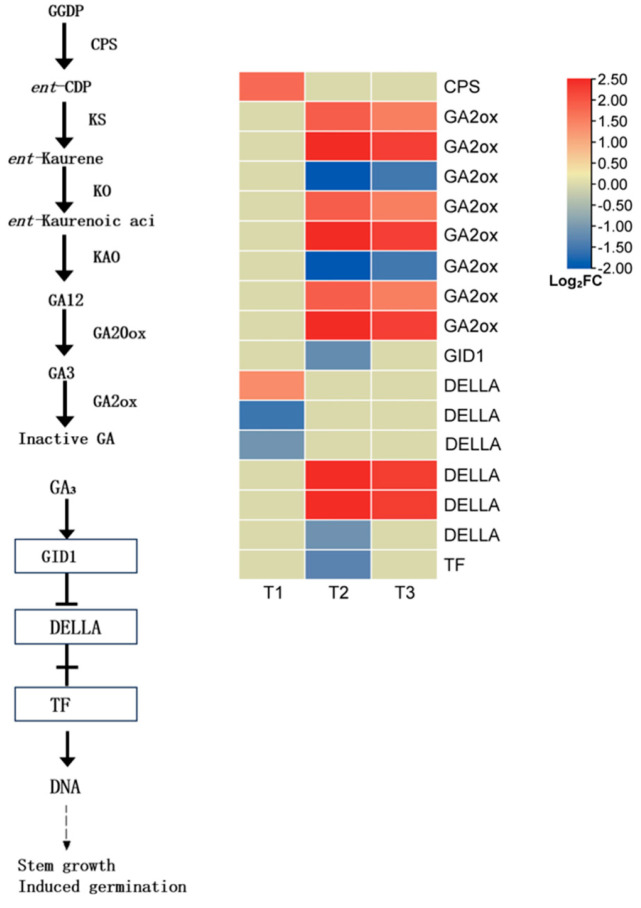
GA biosynthesis and signal transduction pathway in *L. rotatum* during seed germination under GA_3_ treatment.

Gibberellins (GAs) are key phytohormones that promote seed germination, and their biosynthesis and signal transduction pathways play a decisive role in the transition from dormancy to germination. To elucidate the underlying mechanism of GA-regulated germination in *L*. *rotatum*, we analyzed the expression patterns of key genes involved in GA metabolism and signaling (Figure 13).

In the GA biosynthesis pathway, one differentially expressed gene (DEG) encoding copalyl pyrophosphate synthase (*CPS*) was identified, which was significantly upregulated at the T1 stage, indicating early activation of the GA biosynthetic pathway. As a key enzyme in GA deactivation, gibberellin 2-oxidase (*GA2ox*) was represented by six DEGs, all of which were significantly upregulated at T3. This suggests that in the later stage of germination, seeds may enhance the GA deactivation process to maintain hormonal homeostasis and prevent excessive germination.

In the GA signal transduction pathway, the soluble GA receptor *GID1* was significantly downregulated at T2, implying a reduced efficiency of GA signal perception and transduction at this stage. Among the six DEGs encoding the DELLA protein, a known repressor in the GA signaling cascade, two were significantly upregulated and one was downregulated at T2. This indicates that the DELLA-mediated modulation of GA response intensity may be dynamically regulated in response to fluctuating GA levels. The elevated expression of DELLA genes typically reflects suppression of the GA signal, thereby inhibiting downstream physiological responses. In parallel, several transcription factor-related DEGs were also significantly downregulated at T2, further supporting the notion of a weakened GA signaling capacity during the mid-stage of germination.

Overall, *L. rotatum* seeds exhibited early activation of the GA biosynthesis pathway followed by enhanced GA deactivation in the later stage. Meanwhile, GA signaling components showed stage-specific expression patterns. This regulatory pattern likely reflects the dynamic demand and fine-tuned modulation of GA signaling at different germination stages: GA signaling is activated early to break dormancy, while subsequent deactivation and feedback regulation ensure homeostatic control of the germination process.

### 3.6. Relative Expression Analysis of Key Genes

**Figure 14:**
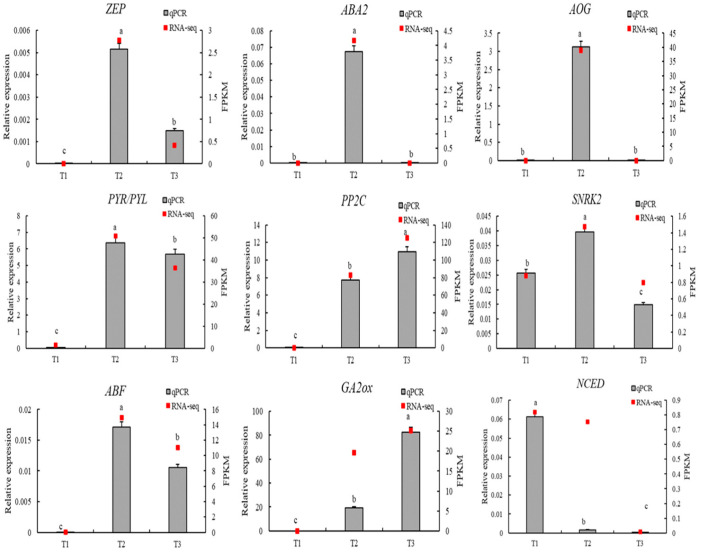
qRT-PCR validation of key hormone-related genes involved in ABA and GA biosynthesis and signaling during seed germination of *L. rotatum*. The lowercase letters (e.g., “a”, “b”, “c”, etc.) are used to indicate statistical differences between the treatment groups. Groups with the same letter are not significantly different from each other, while groups with different letters are significantly different. This method of labeling is commonly used to represent the results of post-hoc statistical tests, such as Tukey’s HSD, following an ANOVA.

To verify the reliability of the RNA sequencing (RNA-seq) data, nine differentially expressed genes (DEGs) closely related to ABA and GA biosynthesis and signaling pathways were selected, including *ZEP*, *NCED*, *ABA2*, *AOG*, *PYR/PYL*, *PP2C*, *SNRK2*, *ABF*, and *GA2ox*. The relative expression levels of these genes were analyzed using qRT-PCR at three germination stages (T1, T2, and T3), and the results were compared with RNA-seq data (Figure 14).

The results showed that the expression trends of most genes were consistent between the two platforms, demonstrating strong correlation and confirming the accuracy and reliability of the RNA-seq data. Among the ABA-related genes, *ZEP*, *PYR/PYL*, *SNRK2*, and *ABF* exhibited significantly upregulated expression at the T2 stage and a decrease at T3, consistent with RNA-seq results. This pattern suggests that ABA signaling is highly active during T2, corresponding to the repression phase prior to germination. *GA2ox*, a key gene involved in GA deactivation, was strongly upregulated at T3, serving as a hallmark of GA signal attenuation in the late germination stage, and its qRT-PCR result fully matched the RNA-seq profile.

Overall, the qRT-PCR validation confirmed the expression patterns observed in the transcriptomic analysis, indicating that the identified DEGs possess high expression stability and research significance. These findings provide strong evidence supporting the hormone-regulated mechanisms underlying seed germination in *L*. *rotatum*.

## 4. Discussion

### 4.1. Effects of GA_3_ Treatment on Seed Germination of L. rotatum

This study demonstrated that the germination performance of *L*. *rotatum* is significantly influenced by both GA_3_ concentration and soaking duration. The optimal treatment condition was identified as 500 mg/L GA_3_ for 24 h, under which the germination percentage reached 77.25%, approximately 37% higher than that of the control. In addition, the germination potential, germination index, and vigor index were all significantly improved under this treatment.

The results indicate a clear concentration-dependent response, with suboptimal performance observed at both lower (100 mg/L) and higher (700 mg/L) concentrations. This suggests the existence of a narrow effective concentration range, a phenomenon that has been documented in other species such as *Annona hybrids* [13] and *Phyllostachys edulis* [14], where excessively high GA_3_ levels induced metabolic stress and reduced viability.

In terms of soaking duration, 24 h was optimal, while prolonged soaking (e.g., 48 h) led to a notable decline in germination metrics. Similar time-dependent sensitivities have been observed in *Chenopodium quinoa* [15], *Hylocereus undatus* [16], and *Prunus domestica* [17], underscoring the importance of precise hormone exposure timing during germination induction.

Importantly, *L. rotatum* seeds exhibited underdeveloped globular embryos prior to treatment, which points to a characteristic of morphological or morphophysiological dormancy. This type of dormancy differs fundamentally from physiological dormancy, in which hormone signaling alone governs germination potential. For seeds with immature embryos, GA_3_ treatment may function not only to modulate hormonal balance but also to promote embryo development and growth competence. Such a dual role of GA has been observed in species with morphological or morphophysiological dormancy, such as *Pinus massoniana* [18], where exogenous GA_3_ enhanced embryo differentiation and shoot meristem activity.

Thus, the favorable response of *L. rotatum* to 500 mg/L GA_3_ treatment likely reflects a combination of accelerated morphological embryo development and hormone-induced germination signaling. The precise mechanisms underlying this response will be further explored through transcriptomic analysis in the following sections.

### 4.2. Transcriptomic Dynamics of L. rotatum Seed Germination

In this study, a time-course transcriptome analysis was conducted at 24 h, 72 h, and 96 h after GA_3_ treatment during seed germination in *L*. *rotatum*. The results revealed a typical time-dependent transcriptional pattern modulated by exogenous gibberellin, with dynamic changes in KEGG pathway enrichment and distinct response modules identified through trend clustering analysis. Such regulatory characteristics have been widely observed in other species, though the specific patterns remain species-dependent.

At the global expression level, the largest number of differentially expressed genes (DEGs; n = 1161) was detected at 72 h post-GA_3_ treatment. This finding is consistent with results from *Phyllostachys edulis*, in which 10 mmol/L GA_3_ significantly enhanced germination activity and respiration rate, accompanied by transcriptomic changes. However, high concentrations of GA_3_ (e.g., 50 mmol/L) induced organelle damage and reduced DEG counts [14], aligning with our observation of DEG reduction and germination inhibition at 700 mg/L GA_3_.

Functionally, DEGs at 24 h were enriched in ribosome and translation-related pathways, while those at 72 h shifted toward phenylpropanoid biosynthesis, hormone signal transduction, and sucrose metabolism. This transition suggests that GA_3_ initially promotes protein translation, followed by reprogramming of structural and hormonal signaling networks. A similar dynamic enrichment pattern was reported in *Pennisetum glaucum*, where germination progressed from dry seeds to swollen embryos and eventually radicle elongation, accompanied by a KEGG enrichment shift from GA signaling to root development [17].

In *Sorghum*, GA_3_ induced expression of nearly 10,000 genes involved in glycolysis, TCA cycle, and hormone-related pathways [19]. Notably, germination repressors such as *DOG1* and *ABI5* were downregulated, highlighting the hormonal antagonism between GA and ABA [20]. The enrichment of “plant hormone signal transduction” at 72 h in *L. rotatum* aligns with findings in *Suaeda glauca* [21] and *Solanum torvum* [22], where *GID1* receptors and *DELLA* repressors were dynamically regulated to activate embryo metabolism and promote cell division [23].

Trend clustering analysis revealed that Cluster 2 and Cluster 3 in *L. rotatum* showed continuous or stage-specific upregulation, significantly enriched in hormone and secondary metabolism pathways such as isoflavonoid, carotenoid, and glutathione metabolism. This pattern is consistent with results in *Fraxinus hupehensis*, where GA_3_ enhanced ROS metabolism, activated antioxidant pathways, and induced expression of genes involved in carbohydrate and flavonoid biosynthesis [24]. Meanwhile, Cluster 5 showed downregulation of primary metabolic pathways, echoing observations in *P. glaucum*, where GA inhibited lipid metabolism to prevent oxidative damage [25], suggesting that *L. rotatum* may modulate energy supply systems in response to GA_3_.

Notably, while core regulatory genes share commonalities across species, expression patterns also differ. In *Triticum aestivum*, genes such as *GA20ox*, *MYB4*, *LEA*, and *CYP78A5* were upregulated and closely associated with seed coat softening and embryo development [26]. In *L. rotatum*, genes within Cluster 1 and Cluster 3 also showed persistent upregulation, including those related to GA biosynthesis and response, indicating a core transcriptional network underlying GA-induced germination. In *S. glauca*, GA treatment upregulated GA biosynthesis and ABA catabolism genes to promote germination [21]. In *Cannabis sativa* and wheat, GA interacted with antioxidant pathways such as SOD and POD, enhancing seed vigor under stress [26,27]. The activation of antioxidant-related modules in *L. rotatum* may also reflect its adaptive mechanism during germination under stress conditions. Recent studies highlight that in species like *Vitis vinifera*, regulatory factors such as *VvMARD* and *VvAGL65*—identified via transcriptome–proteome–hormone integration—participate in dormancy maintenance through ABA and energy metabolism pathways [28]. Likewise, our WGCNA analysis suggests that key modules responsive to GA_3_ may not only reflect hormonal shifts but also underlying embryo developmental progress. Furthermore, *L. rotatum*’s transcriptomic response shares commonalities with rice, where GA–ABA balance, along with ROS and JA, orchestrates the transition from dormancy to germination. In particular, studies show that high dormancy lines in rice exhibit distinct ROS and hormone profiles, with JA–ROS–ABA crosstalk playing a pivotal role. This supports the view that GA_3_ may operate not just through signal transduction but also via redox homeostasis and embryo morphogenesis in seeds with immature embryos [29].

In summary, GA_3_ treatment initiates a tightly regulated, time-dependent transcriptional program in *L. rotatum*, coordinating translation, hormone signaling, and metabolism. The incorporation of findings from grapevine and rice further highlights the need to consider both dormancy type and species-specific regulatory complexity when interpreting germination transcriptomes. The subsequent sections will explore gene-specific regulation and co-expression network features to further dissect this multifaceted response.

### 4.3. Co-Expression Modules and Hormonal Signaling Mechanisms Underlying L. rotatum Seed Germination

In this study, a weighted gene co-expression network analysis (WGCNA) was performed to explore the transcriptional regulation mechanisms underlying *L*. *rotatum* seed germination under GA_3_ treatment. Two key modules were identified based on their strong correlation with hormonal treatments: the Red module was highly associated with the early germination stage (GA_3_-24 h), whereas the Lightcyan module was most strongly correlated with the mid-to-late stage (GA_3_-72 h). Combined with KEGG enrichment and hub gene analysis, these two modules were found to participate in distinct regulatory roles during germination. Moreover, analyses of key genes involved in ABA and GA biosynthesis and signaling revealed highly time-specific expression patterns, suggesting that seed germination in *L. rotatum* is regulated by a multi-layered dynamic network integrating hormone biosynthesis, signal recognition, and feedback regulation.

The Red module exhibited the highest expression at 24 h post-GA_3_ treatment and was significantly enriched in pathways such as steroid biosynthesis, sulfur metabolism, and photosynthesis-antenna proteins, all of which are closely associated with the activation of embryonic cells. Steroid biosynthesis, in particular, involves the production of precursors for growth-promoting hormones such as gibberellins and brassinosteroids, and is essential for the transition of seeds from dormancy to metabolic activation. A similar enrichment of steroid biosynthesis pathways has been reported in *Fraxinus hupehensis*, where GA_3_ treatment promoted cell expansion and elongation [24]. The Red module hub gene, *TRINITY_DN29192_c0_g1*, exhibited the highest network connectivity and may serve as a core regulator in early GA-responsive signaling, warranting further functional validation.

In contrast, the Lightcyan module showed peak expression at 72 h, representing the transcriptional program underlying cell proliferation and metabolic remodeling during the mid-to-late stages of germination. Enriched pathways included benzoxazinoid biosynthesis, autophagy, O-glycan biosynthesis, DNA replication, and mismatch repair, indicating an active mitotic phase accompanied by intensive metabolic reprogramming and structural renewal. Similar expression patterns have been observed in *S. glauca* and *F. hupehensis*, where DNA replication and hormone-related pathways were significantly enriched at 72 h following GA_3_ treatment, marking the transition from perception/preparation to cellular reorganization [21,24]. The Lightcyan hub gene, *TRINITY_DN27159_c0_g1*, formed a radiating co-expression network with multiple metabolic and signaling genes, suggesting that it plays a central role in maintaining cellular state transitions.

In terms of hormone signaling, ABA biosynthesis genes such as *ZEP* and *NCED* were generally downregulated from T1 to T2, while components of the ABA signaling cascade, including *PYL* receptors, *SnRK2* kinases, and *ABF* transcription factors, were significantly upregulated at T3. This indicates a “biosynthesis suppression–signaling activation” model in which ABA initially functions as a dormancy-maintaining factor but later becomes re-engaged in coordinating embryo growth. Similar temporal regulation of ABA signaling has been reported in *Bletilla striata* and *Benincasa hispida*, supporting the antagonistic–coordinated model between GA and ABA in the shift from dormancy release to radicle elongation [30,31].

With respect to GA signaling, the expression of *CPS* was upregulated at T1, indicating early activation of GA biosynthesis, while the late-stage upregulation of *GA2ox* at T3 suggests enhanced GA deactivation to avoid hormonal overaccumulation and metabolic imbalance. This “synthesis–feedback” pattern has also been observed in *Pistacia chinensis* and *Saposhnikovia divaricata*, where fine-tuned GA signaling is critical for radicle emergence and growth homeostasis [32,33]. Furthermore, expression of *GID1* was downregulated at T2, suggesting a feedback-sensitive GA perception system during the mid-stage. Meanwhile, *DELLA* proteins exhibited dynamic expression patterns. As negative regulators of GA signaling, increased *DELLA* expression likely reflects the system’s need to moderate signal intensity during peak GA activity.

In summary, *L. rotatum* seeds exhibit distinct stage-specific expression patterns and multi-layered regulatory networks in response to GA_3_ treatment. The early phase is characterized by the Red module, which activates hormone signaling and primary metabolic pathways, while the Lightcyan module dominates the mid-to-late phase, promoting cell cycle progression and metabolic restructuring. ABA and GA signaling pathways interact dynamically throughout germination, with one downregulated and the other activated at specific stages. By integrating WGCNA modules, hub genes, enriched pathways, and hormone expression profiles, this study provides new insights into the molecular mechanisms underlying legume seed germination and offers theoretical support for precision hormone regulation strategies in seed biology.

## 5. Conclusions

This study systematically elucidated the promotive effects of exogenous gibberellin (GA_3_) on seed germination of *L*. *rotatum* and its underlying transcriptional regulation. Treatment with 500 mg/L GA_3_ for 24 h significantly enhanced germination rate, vigor index, and seed uniformity, representing the optimal condition.

Transcriptomic profiling revealed time-dependent gene expression patterns under GA_3_ treatment. K-means clustering and KEGG enrichment indicated a shift from early activation of translation and energy mobilization to later enrichment in hormone signaling, DNA replication, and cell cycle control.

WGCNA identified two key modules, Red and Lightcyan, associated with early and late germination phases, respectively. These modules were enriched in pathways including hormone biosynthesis, autophagy, and cell cycle regulation, and contained hub genes potentially critical for GA_3_ response.

Hormonal analysis demonstrated a phased antagonism between ABA and GA. ABA biosynthesis was suppressed in early stages, with signal transduction reactivated later; GA biosynthesis was induced early and inactivated via *GA2ox* in later stages. The dynamic expression of *GID1* and *DELLA* further revealed the complexity of GA signal modulation during germination.

## Figures and Tables

**Table 1:** Statistical results of *L. rotatum* seed sequencing.

ID	Read Number	Base Number	GC Content	% ≥ Q30
GA_3_-24h1	20,232,799	6,055,885,947	43.44%	94.05%
GA_3_-24h2	20,522,890	6,143,199,636	43.66%	94.28%
GA_3_-24h3	19,679,395	5,891,520,830	43.58%	94.33%
GA_3_-72h1	19,221,648	5,754,433,917	42.65%	94.10%
GA_3_-72h2	20,317,439	6,076,379,889	42.87%	94.63%
GA_3_-72h3	19,556,790	5,847,748,648	42.77%	94.62%
GA_3_-96h1	20,051,187	6,003,725,668	42.55%	94.29%
GA_3_-96h2	20,051,334	6,002,314,071	42.56%	94.27%
GA_3_-96h3	20,035,098	5,996,430,036	42.58%	94.54%
H_2_O-24h1	19,906,509	5,955,136,968	43.88%	94.12%
H_2_O-24h2	22,168,127	6,632,734,420	43.90%	94.75%
H_2_O-24h3	19,938,952	5,969,888,304	43.81%	93.93%
H_2_O-72h1	20,582,702	6,157,794,233	43.21%	94.67%
H_2_O-72h2	20,383,386	6,098,756,120	42.97%	94.89%
H_2_O-72h3	20,312,823	6,075,064,831	43.18%	94.39%
H_2_O-96h1	20,237,393	6,057,775,075	42.77%	94.46%
H_2_O-96h2	20,698,983	6,196,232,376	43.03%	94.20%
H_2_O-96h3	20,241,326	6,060,140,013	42.83%	94.16%

## Data Availability

Due to the confidentiality of the project, the relevant data cannot be made publicly available at this time. The data will be shared in accordance with the relevant regulations at an appropriate time.

## Supplementary Materials

The following supporting information can be downloaded at: https://www.mdpi.com/article/10.3390/genes16080878/s1, Table S1. qRT-PCR primer information.
